# The Commercial Antibodies Widely Used to Measure H3 K56 Acetylation Are Non-Specific in Human and *Drosophila* Cells

**DOI:** 10.1371/journal.pone.0155409

**Published:** 2016-05-17

**Authors:** Sangita Pal, Hillary Graves, Ryosuke Ohsawa, Ting-hsiang Huang, Pingping Wang, Laura Harmacek, Jessica Tyler

**Affiliations:** 1 Department of Pathology and Laboratory Medicine, Weill Cornell Medicine, 1300 York Avenue, New York, New York, 10065, United States of America; 2 Department of Epigenetics and Molecular Carcinogenesis, University of Texas MD Anderson Cancer Center, Houston, Texas, 77030, United States of America; 3 Department of Cell Biology, University of Colorado School of Medicine, Aurora, Colorado, United States of America; Texas A&M University, UNITED STATES

## Abstract

Much of our understanding of the function of histone post-translational modifications in metazoans is inferred from their genomic localization and / or extrapolated from yeast studies. For example, acetylation of histone H3 lysine 56 (H3 K56Ac) is assumed to be important for transcriptional regulation in metazoan cells based on its occurrence at promoters and its function in yeast. Here we directly assess the function of H3 K56Ac during chromatin disassembly from gene regulatory regions during transcriptional induction in human cells by using mutations that either mimic or prevent H3 K56Ac. Although there is rapid histone H3 disassembly during induction of some estrogen receptor responsive genes, depletion of the histone chaperone ASF1A/B, which is required for H3 K56 acetylation, has no effect on chromatin disassembly at these regions. During the course of this work, we found that all the commercially available antibodies to H3 K56Ac are non-specific in human cells and in *Drosophila*. We used H3-YFP fusions to show that the H3 K56Q mutation can promote chromatin disassembly from regulatory regions of some estrogen responsive genes in the context of transcriptional induction. However, neither the H3 K56R nor K56Q mutation significantly altered chromatin disassembly dynamics by FRAP analysis. These results indicate that unlike the situation in yeast, human cells do not use H3 K56Ac to promote chromatin disassembly from regulatory regions or from the genome in general. Furthermore, our work highlights the need for rigorous characterization of the specificity of antibodies to histone post-translational modifications *in vivo*.

## Introduction

Chromatin is the physiological template for all genomic processes in Eukaryotes. The basic repeating unit of chromatin, the nucleosome, consists of 146bp of DNA wound 1.75 times around two molecules of each of the histones H3, H4, H2A and H2B [1]. Nucleosomes decorate our entire genome, enabling the genetic material to be packaged and protected within the cell. Studies in the single celled eukaryote budding yeast have made it clear that the histone components of chromatin are removed from the DNA to enable transcription, DNA repair and replication to occur [2, 3]. Chromatin disassembly enables the machinery that mediates these genomic processes to gain intimate access to the DNA in order to perform their function. How histones are removed from the DNA during these processes is partly understood in yeast, and involves histone chaperone proteins that bind stoichiometrically to the histones in order to remove them from the DNA [4]. Removal of histones from the DNA is also helped by histone post-translational modifications (PTMs) that weaken histone-DNA interactions, such as acetylation of H3 lysine 56 (H3 K56Ac) [5, 6][7]. Whether similar mechanisms are at play during genomic processes in metazaons is unclear.

The study of the function of histone PTMs is facile in yeast, because it is easy to mutate the histone encoding genes given that there are only two copies of each, and then look at the resulting effects. However, there are hundreds of genes encoding each canonical histone in metazoan cells [8]. As a consequence, the majority of what we know about histone PTMs in mammalian cells comes from correlation studies that examine the localization of histone PTMs along the genome [9]. However, these analyses do not test functional roles of the histone PTMs, and such correlative studies are intimately dependent on the specificity of the histone PTM antibodies.

Histone H3 K56Ac has been identified in metazaons by mass spectrometry [10] [11]. Acetylation of H3 K56Ac in humans is mediated by the histone acetyl transferases CBP and p300 in concert with the histone chaperone ASF1. Chromatin immunoprecipitation (ChIP) analyses of the localization of H3 K56Ac in mammalian cells first found it to be associated with the regulatory regions of pluripotency genes in stem cells [10], implicating it in transcriptional regulation. As such, much of the inferred role of H3 K56Ac in transcription in metazoan cells comes from studies with antibodies to H3 K56Ac, which show H3 K56Ac localizing to active promoters or enhancers in mammalian cells [10, 12–21]. However, in contrast to yeast, where all newly synthesized histones are acetylated on H3 K56Ac, the abundance of H3 K56Ac in human cells is extremely low, approximately 0.03% of all histones, as determined by mass spectrometry analysis [22]. As such, direct extrapolation of the function of H3 K56Ac from yeast to humans may not be accurate. These papers imply that H3 K56Ac plays a role in transcription in metazoans, based on its localization patterns. However, this has not been tested functionally. Mutation studies in yeast have shown that H3 K56Ac promotes transcriptional activation in yeast by enabling histones to be more readily removed from promoter regions [7]. Therefore, we set out to examine whether H3 K56Ac does indeed play a role in transcription, via a potential function in promoting histone removal from regulatory sequences of human genes. We find that histone disassembly accompanies induction of some estrogen-responsive genes, and that this is accompanied by an apparent increase in H3 K56Ac. However, inactivation of ASF1 to block H3 K56Ac has no effect on chromatin disassembly or gene induction, indicating that H3 K56Ac is not used in human cells to promote chromatin disassembly from these genes. We utilized YFP fused to H3 to show that mutation of K56 to glutamine (Q) to mimic acetylation promotes chromatin disassembly from regulatory regions during transcriptional induction. However, using FRAP analysis, we found that mutations that block or mimic H3 K56Ac have no influence on chromatin disassembly from the bulk genome in human cells. During the course of our studies, we found that all of the commercially available H3 K56Ac antibodies are non-specific in westerns and ChIP analyses in metazoan cells, recognizing other acetylated lysines on histone H3. As such, studies using H3 K56Ac antibodies in metazoans should be interpreted with caution.

## Results and Discussion

### Acetylation of H3 K56Ac is not required for chromatin disassembly from some estrogen-responsive gene regulatory regions

**Fig 1:**
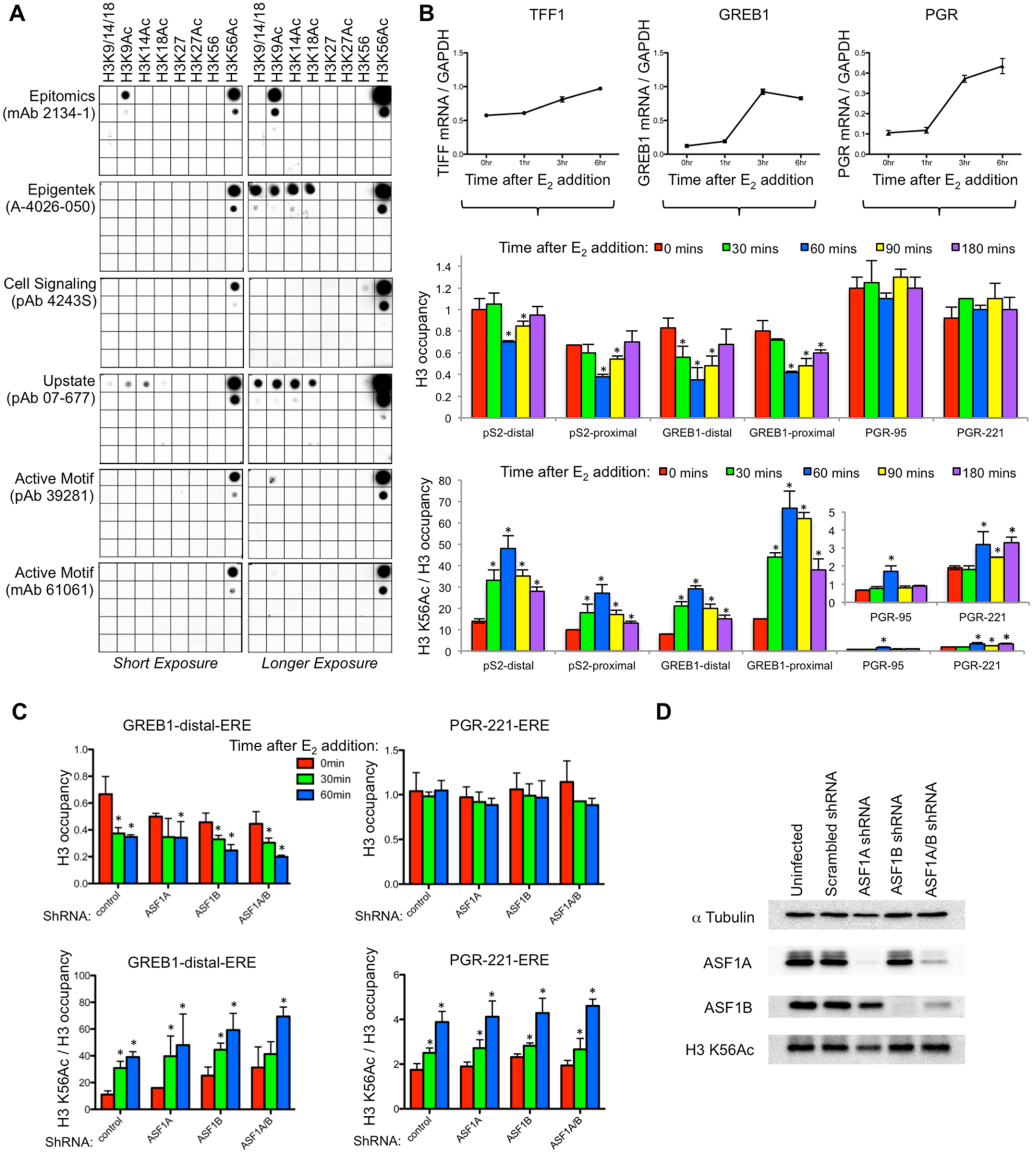
H3 K56Ac is not required for chromatin disassembly gene regulatory regions of estrogen responsive genes. **A**. Dot blot analysis of the indicated commercial H3 K56Ac antibodies, the indicated peptides. 10 μl of each peptide at 300 μM concentration was spotted on the top row, followed by 10 fold dilutions below. A short and longer exposure are shown. **B**. The top shows RT-PCR analysis of mRNA induction at the indicated times after addition of estradiol, normalized to GAPDH. Data are the average and standard deviation of three independent experiments. Below is shown a ChIP analysis of histone H3 occupancy at the indicated time points after estradiol addition from the same time course as the mRNA analysis, at the indicated EREs in the pS2 (*TFF1)*, *GREB1*, and *PGR* promoters. Each data point was normalized to the input and a telomeric control region at the same time point. At the bottom is shown a ChIP analysis of histone H3 K56Ac levels normalized to H3 occupancy. Each data point was normalized to the input and a telomeric control region at the same time point. Shown are the average and standard deviation of three independent experiments. The H3 K56Ac data for the *PGR* gene regions are shown again in the inset with the y-axis expanded, to enable visualization of the very low signal. * indicates significant changes from time 0, p<0.05 measured by the Student’s unpaired t-test. **C**. ChIP analysis of H3 and H3 K56Ac levels normalized as in B, during the indicated shRNA knockdowns. Shown are the average and standard deviation of three independent experiments. * indicates significant changes from time 0, p<0.05 measured by the Student’s unpaired t-test. **D**. Western blot analysis of ASF1A, ASF1B, H3K56 Acetylation, and tubulin alpha from samples taken from the experiment shown in C. Protein extracts were made with RIPA buffer from MCF7 cells that were infected with lentivirus of non-silencing (control), ASF1A shRNA, ASF1B shRNA, ASF1A/B shRNA. The Active Motif polyclonal antibody was used for experiments shown in B-D.

Induction of some ER-responsive genes in humans is accompanied by transient chromatin disassembly from gene regulatory regions, but this does not require H3 K56 acetylation. In order to analyze potential roles of H3 K56Ac during transcription in mammalian cells, we examined some of the commonly studied estrogen-responsive genes in MCF7 cells. Because our earlier studies in yeast had indicated that H3 K56Ac drives chromatin disassembly [7], we asked whether H3 K56Ac levels increase at these genes during transcriptional induction and whether this was required for chromatin disassembly from promoters. Due to previous concerns raised about the specificity of some commercially available antibodies to H3 K56Ac [22], we tested specificity of the available commercial antibodies to H3 K56Ac. On a dot blot, in addition to recognizing a peptide carrying H3 K56Ac, the Epitomics antibody also recognized an H3 K9Ac peptide (Fig 1A), even though earlier supplies of this antibody were highly specific, as shown by loss of all signal in western analysis upon mutation of the K56 residue to block acetylation in yeast or human cells [10] [11]. Why the stringency of the Epitomics H3 K56Ac antibody has become less specific over time is unclear. Other H3 K56Ac antibodies from Upstate and Epigentek also recognized multiple other acetylated lysines from the N-terminus of H3 in addition to H3 K56Ac (Fig 1A). By dot blot, the most specific antibodies were those from Cell Signalling and Active Motif, so we continued to use those antibodies for our analyses of H3 K56Ac levels in human promoter regions.

Following addition of estradiol, we observed significant increases of estrogen-responsive gene transcripts such as *TFF1*, *PGR*, and *GREB1* (Fig 1B). We examined histone H3 occupancy and H3 K56Ac in the vicinity of the estrogen receptor (ER) binding sites, also known as Estrogen response elements (EREs), during induction of gene expression [23]. We specifically used H3 in order to measure chromatin disassembly from the promoter, because H3 is a central component of the nucleosome, such that a reduction in H3 indicates a reduction in nucleosome level. H3 and H4 are obligate heterodimers, meaning that we had no need to examine H4 occupancy. We did not examine H2A/H2B because they are highly dynamic and can be removed from the DNA without the need for complete nucleosome disassembly. We found significant histone H3 disassembly from the pS2 promoter of the *TFF1* and *GREB1* genes, but not from the *PGR* gene, at 60 minutes after addition of estradiol (Fig 1B). The reason why chromatin disassembly occurs from some estradiol-regulated promoters, but not from others, is not clear. The chromatin disassembly was followed by a subsequent progressive return of the H3 to the DNA at 90 and 180 minutes (Fig 1B). Although we were examining the steady state RNA level, a recent gro-seq analysis of nascent RNA indicates the transient nature of E2 induced transcription [24], consistent with our observed chromatin reassembly at later times after adding estradiol. These results show that the induction of some, but not all, of the estrogen responsive genes is accompanied by transient promoter nucleosome disassembly. The transient nature of histone removal during induction of human genes has been observed previously at the interleukin-2 promoter [25].

ChIP analysis of H3 K56Ac with the antibodies all showed similar results, and results for the Active Motif polyclonal antibody are shown in here. The signal from the H3 K56Ac antisera significantly increased at all three estrogen responsive promoters upon gene induction, peaking at 60 minutes (Fig 1B), while there was no change in H3 K56Ac at control regions (S1 Fig). The increase and decrease in the H3 K56Ac signal was inversely proportional to the removal and return of histone H3 at the pS2 promoter of the *TFF1* gene and at the *GREB1* promoter, but also the same timing of H3 K56Ac increase and decrease was seen at the *PGR* promoter that does not undergo chromatin disassembly during gene induction.

We next asked whether H3 K56Ac is responsible for histone removal from gene regulatory regions during the estrogen response. The HAT, CBP, plays multiple roles during transcription so we chose not to deplete H3 K56Ac via CBP knockdown. Instead, we knocked down the histone chaperone ASF1A that is required for CBP-mediated H3 K56 acetylation [11, 26]. ASF1A also has a homolog ASF1B that is not significantly involved in promoting H3 K56Ac in vivo [11, 26]. Upon knocking down either ASF1A, ASF1B or both ASF1A and ASF1B, we observed no significant disruption in the extent or kinetics of chromatin disassembly from the regulatory regions of the the *TFF1* and *GREB1* genes (Fig 1C and S2 Fig). This result indicates that H3 K56Ac is not required for chromatin disassembly from these human promoters. We also saw no defect in induction of transcription from the estrogen responsive genes upon ASF1 knockdown (S3 Fig). Strikingly, when we examined the occupancy of H3 K56Ac by ChIP analyses, we found that the H3 K56Ac occupancy was unchanged by ASF1 knockdown (Fig 1C and S2 Fig). The knockdowns of ASF1A and ASF1B were relatively efficient (Fig 1D), yet we also still detected H3 K56Ac by western blot upon ASF1 knockdown. These results suggested that the H3 K56Ac antisera may be recognizing other H3 acetylations by western and ChIP analysis, in addition to H3 K56Ac. This led us to retest the specificity of the H3 K56Ac antibodies more stringently, given that ASF1A is required for acetylation of H3 K56 *in vivo* [11].

### Commerically available H3 K56Ac antibodies are non-specific in mammalian cells and in fly tissues

**Fig 2:**
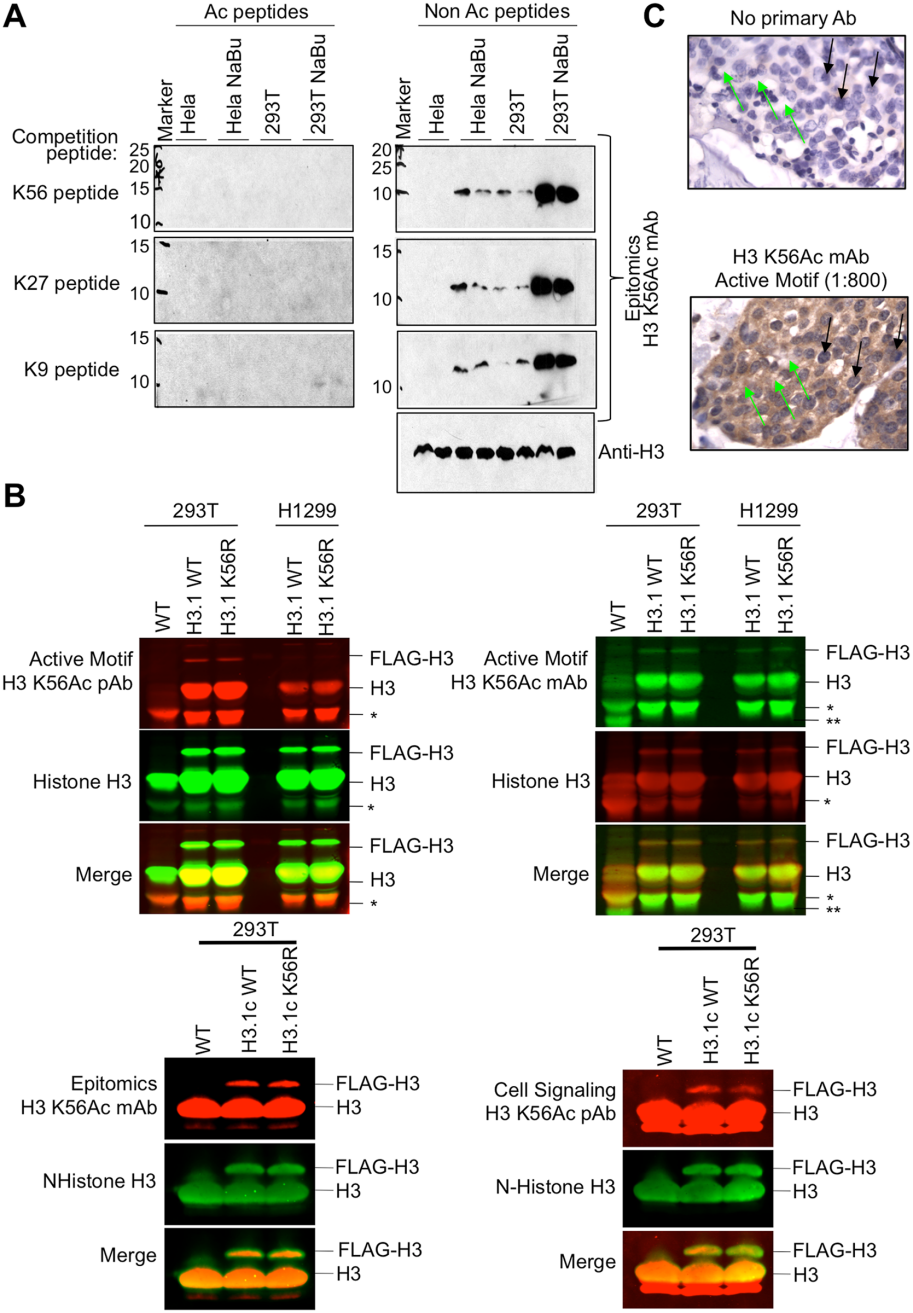
Commercial H3 K56Ac antibodies are non-specific in human cells. **A.** Peptide competition analysis using the indicated peptides, of acid extracted histones from HeLa and 293 cells that were treated with or without sodium butyrate (NaBu) to inhibit class I and II HDACs. The antibody used in the western is indicated on the right. Duplicate samples are loaded for each condition. The sizes on the left indicate kD size of Biorad Dual Color Standards molecular weight markers that were transposed onto the film from the membrane. **B**. Western blot analysis of acid extracted histones from 293 cells that were wild type (WT) or stably expressed H3.1c-FLAG or H3.1c K56R-FLAG, or from H1299 cells that stably expressed H3.1c-FLAG or H3.1c K56R-FLAG. * indicates a proteolytic degradation product of H3 generated by cleavage of the N-terminus that occurs upon generating the total protein extracts. ** indicates a smaller H3 degradation product seen in the wild type sample only, due to over handling of the extract. Data is shown for the Active Motif, the Epitomics and the Cell signalling H3 K56Ac antibodies. **C**. IHC analysis of the Active Motif mAb, on breast cancer tissue samples shows non-specific cytoplasmic staining instead of distinct nuclear staining. Green arrows point to cytoplasm and black arrows point to nuclei. Both slides are also stained with haematoxylin and eosin (H&E) stain, which stains nuclei blue and eosinophilic structures pink.

To rigorously test the specificity of the H3 K56Ac antibodies within the context of other cellular proteins, we used multiple approaches. First, we performed peptide competition analyses of western blots, by prebinding the H3 K56Ac antibodies with peptides that carried either no acetylation or acetylation of H3 K9, K27, or K56. Noteworthy, all the antibodies only recognized a single band by western blot that was the size of histone H3, indicating that they are all seemingly specific for histone H3. Furthermore, all of the H3 K56Ac antibodies tested here recognized more H3 upon treatment with HDAC inhibitors, indicating that they are recognizing acetylated H3. However this is unlikely to be H3 K56Ac, because it has been previously shown by mass spectrometry analysis that treatment with these same HDAC inhibitors does not increase the levels of H3 K56Ac [22]. We found that the Epitomics antibody could be competed away by peptides carrying K27Ac and K9Ac as well as K56Ac (Fig 2A), while the Cell Signalling antibody could be competed away with K27Ac and K56Ac (S4 Fig). Similar results have been shown with the Epitomics and Active Motif antibodies in the same kind of assay previously [22].

The peptide competition experiments use levels of competing acetylated peptide far in excess of the level of the H3 K56Ac protein on the membrane. Therefore, we analyzed the selectivity of the antibody in a situation in which all the different acetylated lysines would be present in the physiologically relevant ratios found within the cell, i.e. in a crude protein extract. To stringently test the specificity of the H3 K56Ac antibodies *in vivo*, we used mammalian H1299 and 293T cell lines carrying stably integrated FLAG-tagged H3.1, that was either wild type or had K56 mutated to R to prevent it being acetylated. We had to use acid extracted histones to isolate enough protein to be detected by western blot, given that the FLAG-tagged H3 was only expressed to about 5% the level of endogenous histones. Histone H3 is highly prone to proteolytic cleavage / degradation during the histone isolation, as is seen in particular in the wild type cells (marked by the asterisks in Fig 2B). Regardless of the H3 degradation, the purpose of including the wild type cells was to show that the H3 K56Ac antibody was not recognizing an irrelevant background band. Strikingly, we found that all the commercial H3 K56Ac antibodies recognized the H3 K56R-FLAG tagged protein as effectively as the wild type H3-FLAG (Fig 2B), even though K56R is unable to be acetylated on this residue. This was the case for the H3 K56Ac antibodies from Epitomics, Cell Signalling and Active Motif (Fig 2B), indicating that these H3 K56Ac antibodies all recognize H3 in the absence of H3 K56Ac. We tried numerous different dilutions of the antibodies to try to find a dilution at which the antibodies show specificity for H3 K56Ac and at all dilutions they recognized H3 K56R-FLAG and wild type H3-FLAG equally well. We also tested the ability of the H3 K56Ac antibodies to recognize MCF7 cells transfected with YFP-tagged H3.3 that was either wild type, or had K56 mutated to Q or R. The H3 K56Ac antibodies recognized H3.3-YFP irrespective of whether amino acid 56 was a K,R or Q, with the H3 K56Ac signal corresponding to the expression level of H3.3-YFP (S5A Fig).

We also tested the ability of the Active Motif mAb antibody to recognize nuclear proteins by immuno histochemistry (IHC) (Fig 2C and S5B Fig) and found it to recognize significant amounts of cytoplasmic proteins at all tested dilutions. It is possible that the cytoplasmic signal in IHC is from acetylated histone H3, given that acetylation of H3 on lysine 14 or lysine 18 occurs in the cytoplasm [27], but it is unlikely to be recognizing H3 K56Ac in the cytoplasm given that this acetylation event is mediated by p300 and CBP [11] presumably in the nucleus. Noteworthy, this H3 K56Ac antibody is not advertised as being validated for IHC. However, it is advertised as being validated for ChIP, which uses a similar mode of recognition of native epitopes and both methods use fixation with 3.7% formaldehyde.

**Fig 3:**
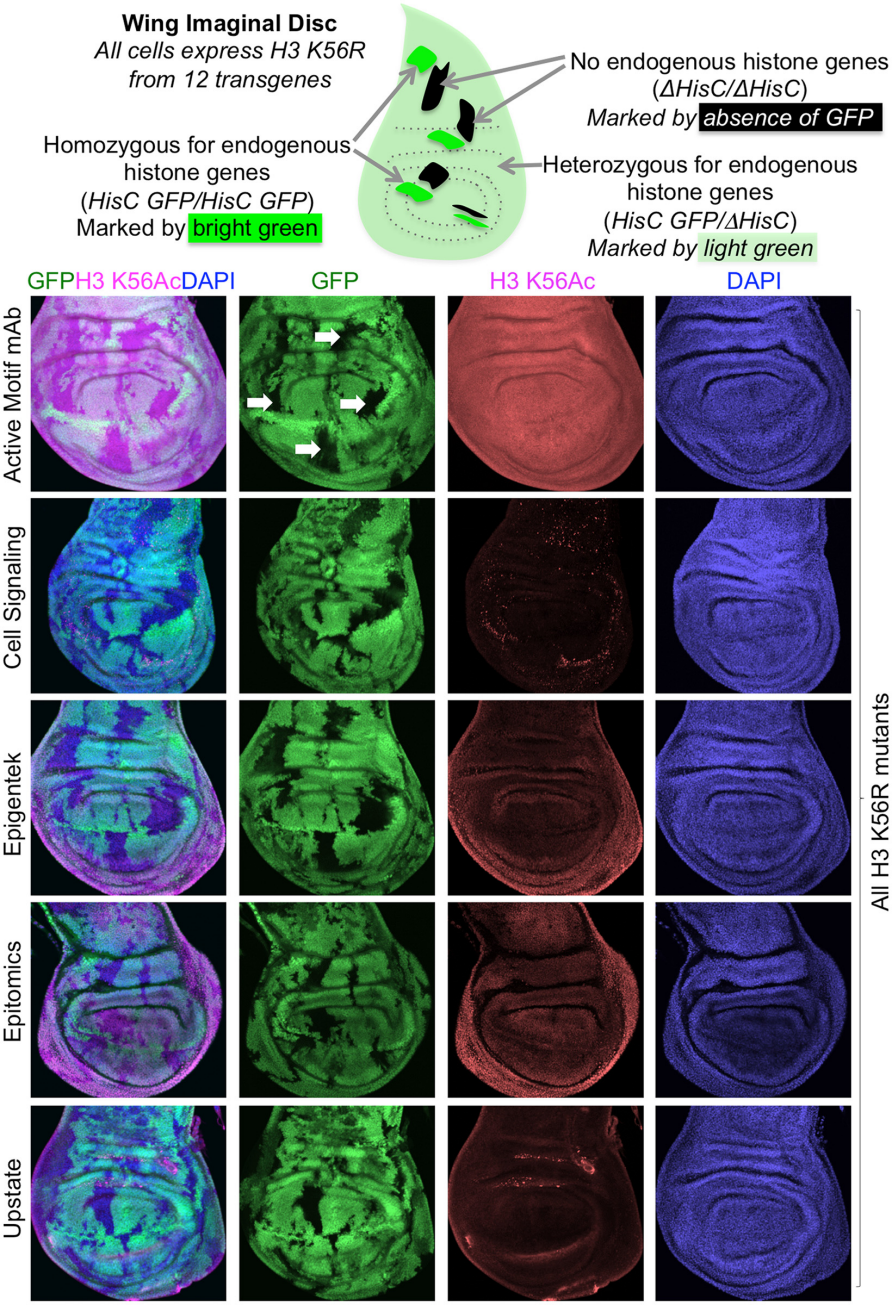
Commercial H3 K56Ac antibodies are non specific in flies. The top schematic explains the mosaic nature of the wild type histone expression, indicated by GFP staining. All cells express H3 K56R, but the black patches express no endogenous wild type histones. Below are shown wing imaginal discs from flies with clones of cells expressing only H3 K56R (indicated by white arrows in the top row) that are marked by lack of GFP expression. Staining with the indicated H3 K56Ac antibodies showed no difference between H3 K56R mutant clones or the adjacent wild type clones, indicating that the antibodies are non specific. Representative images are shown.

The sequences around H3 K56 are absolutely conserved through evolution. Therefore, as a final test of specificity, we examined the ability of all of the H3 K56Ac antibodies to recognize H3 K56Ac in fly tissues where all histone H3 has K56 mutated to R to prevent acetylation [28]. To do this, we used a system that enables deletion of both copies of the endogenous histone gene locus *HisC* (each containing approximately 400 genes expressing the canonical core histones) while at the same time also supplying 12 copies of mutant histone genes on transgenes [29, 30]. Flies where H3 K56R is the only form of histone H3 are inviable so we had to generate mosaic flies [28]. To do this, we made flies that carried 12 copies of the H3 K56R transgenes in every cell, and were heterozygous for deletion of the histone gene locus, *HisC*. We then induced a tissue specific flippase in the wing disc during development to cause recombinational exchange of the left arm of chromosome II carrying *HisC*, which is also marked by GFP (S6 Fig). Subsequent growth of the cells results in wing discs with patches (clones) of cells that are bright green, having gained an extra copy of the *HisC* locus expressing endogenous histones and an extra copy of GFP (Fig 3). The recombination event also results in patches of cells (clones) that have no green signal, having lost the *HisC* locus and so express neither endogenous histones nor GFP (Fig 3 and S6 Fig). The remainder of the cells are heterozygous and are light green, having one copy of *HisC* and one copy of GFP. If the H3 K56Ac antibodies were recognizing only H3 K56Ac, one would expect to see loss of signal in the black clones that express no wild type histones. However, for all the H3 K56Ac antibodies, we observed no change in the intensity of signal in the black clones expressing only H3 K56R as compared to the adjacent green regions expressing wild type H3 (Fig 3). Multiple different dilutions of antibody were used, unsucessfully, to try to find a dilution that showed specificity. As such, the H3 K56Ac antibodies show non-specificity in flies.

Taken together, these data indicate that at least in our hands, we were unable to achieve specific recognition of H3 K56Ac in flies or human cells with any of the commercially available H3 K56Ac antibodies, via ChIP, immunofluorescence, IHC or western analyses. The non-specificity of the H3 K56Ac antibodies would explain the confusion as to the identity of the responsible histone deacetylase (HDAC) in mammalian cells, because SIRT1, SIRT2, SIRT6, HDAC1, and HDAC2 have each been reported to be the H3 K56Ac HDAC [11, 31–39]. Similarly, the non-specificity of these antibodies could explain the contradictory reports of the occurrence of H3 K56Ac during the cell cycle and DNA repair [11, 31, 40–48]. As such, data obtained with commercial H3 K56Ac antibodies in metazoan cells, are likely to be largely due to recognition of additional acetylated lysines on H3, and should be interpreted with caution.

### Mutations that mimic persistent H3 K56Ac can promote histone disassembly during gene induction

**Fig 4:**
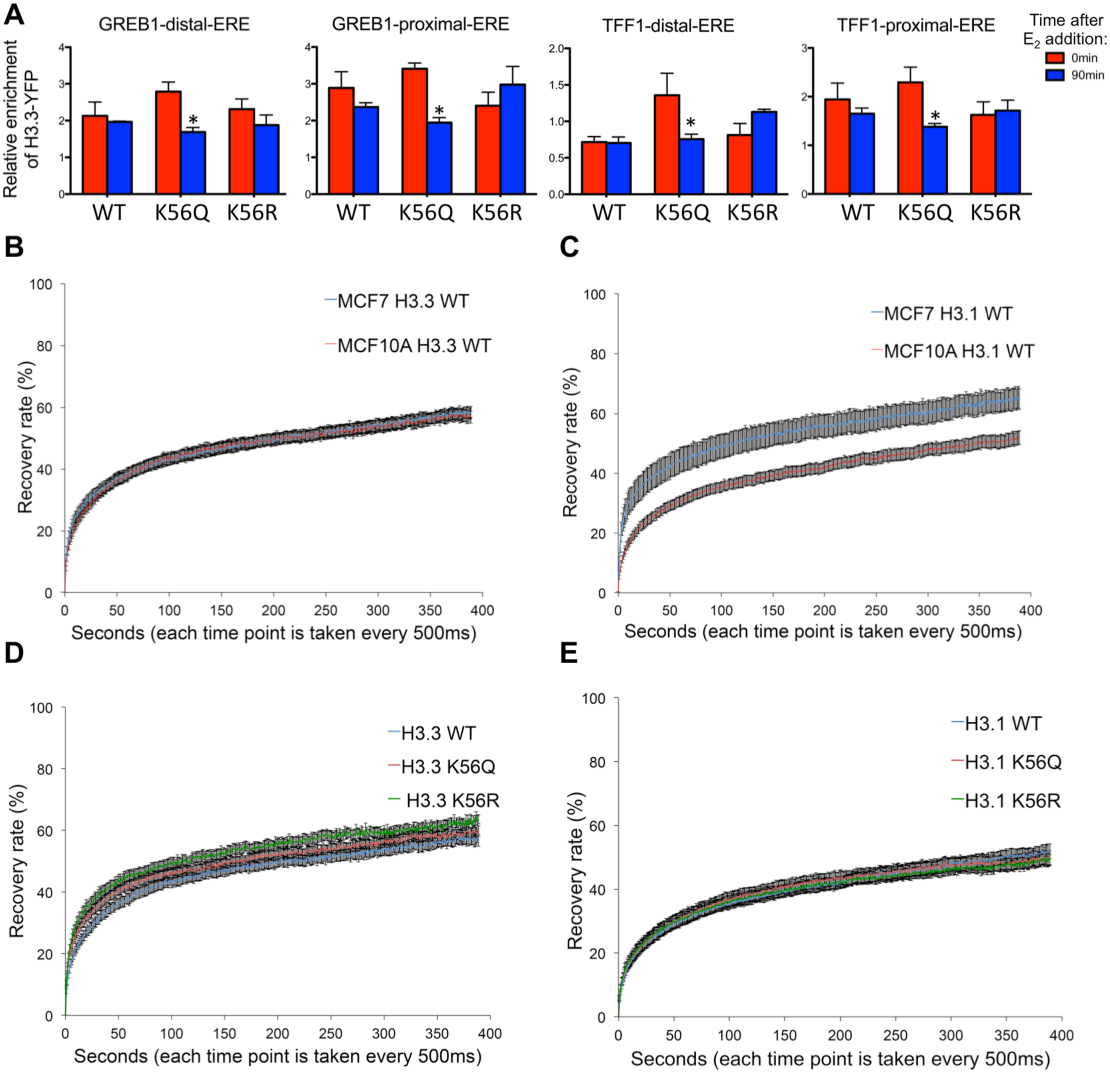
Mutation mimicking H3 K56Ac can promote histone disassembly from promoters during their induction, but not from bulk chromatin. **A.** ChIP analysis of YFP from cells expressing wild type H3.3-YFP or H3.3 K56Q-YFP or H3.3 K56R-YFP in MCF7 cells following addition of estradiol. Data are normalized as in Fig 1. Data are the average and standard deviation of three independent experiments. * indicates significant changes from time 0, p<0.01 measured by the Student’s unpaired t-test **B**. FRAP analysis of YFP tagged H3.3 in MCF7 vs MCF10A cells (average and SD are shown for 30 cells). **C**. FRAP analysis of YFP tagged H3.1 in MCF7 vs MCF10A cells (average and SD are shown for 30 cells). **D**. FRAP analysis of H3.3-YFP or H3.3 K56Q-YFP or H3.3 K56R-YFP in MCF7 cells. **E**. FRAP analysis of H3.1-YFP or H3.1 K56Q-YFP or H3.1 K56R-YFP in MCF7 cells.

In order to analyze the influence of H3 K56Ac on chromatin disassembly without having to use H3 K56Ac antibodies, we transfected YFP-tagged constructs containing H3.3 K56Q, to mimic acetylation, K56R to prevent acetylation and wild type H3.3 into MCF7 cells. By YFP ChIP analysis, we found that H3.3 K56Q-YFP was preferentially removed from the regulatory regions of *TFF1* and *GREB1* as compared to the wild type H3.3-YFP and H3.3 K56R-YFP during gene induction (Fig 4A). All data are shown normalized to a control region of the genome, where there was no significant change in the H3.3-YFP occupancy upon addition of estradiol. The expression level of H3.3 K56R-YFP was equivalent to wild type H3.3-YFP (S5A Fig), yet the K56R mutation had no effect on chromatin disassembly of H3.3-YFP (Fig 4A). The level of expression of H3.3 K56Q-YFP was consistently about half of that of wild type H3.3-YFP (S5A Fig). However, because these experiments are comparing H3.3 K56Q-YFP occupancy to H3.3 K56Q-YFP occupancy at different time points after addition of estradiol, the difference in expression of H3.3 K56Q-YFP and wild type H3.3-YFP is irrelevant. The H3.3-YFP proteins were expressed to 5–10% of the endogenous histone level, and therefore as expected, had no dominant effect on induction of gene expression or bulk histone occupancy. These analyses have the caveat that mutation of H3 K56 will also affect other post-translational modifications, including methylation [14, 16]. However, this is not an issue for us, because the H3 K56R mutant had no effect in our experiments, indicating that neither H3 K56 methylation nor acetylation is relevant for chromatin disassembly at the human promoters we examined. Taken together, these data indicate that while H3 K56Ac is not required for chromatin disassembly (Fig 1C), a mutation mimicking persistent acetylation of H3 K56 can promote chromatin disassembly (Fig 4A). These data can be reconciled with the fact that the level of naturally occurring H3 K56Ac is so low in mammalian cells that there is likely insufficient amounts of it to promote significant amounts of chromatin disassembly. Presumably, mammalian cells utilize other mechanisms to drive chromatin disassembly from promoter regions during gene induction, and this awaits further investigation.

### Mutations that mimic or prevent constitutive H3 K56 have no effect on histone dynamics globally

To determine whether the ability of H3 K56Q to promote chromatin disassembly was specific to genes undergoing transcriptional induction, or was a global phenomena, we performed fluorescence recovery after photobleaching (FRAP) of YFP-H3. After photobleaching approximately 1/4 of the cell (S7A Fig), fluorescence recovery is thought to be due to bleached histones dissociating from the DNA being replaced by unbleached histones binding to the DNA. If the histone mutation promotes chromatin disassembly, it would be expected to lead to faster recovery after photobleaching. Exchange of the soluble pool of rapidly diffusing proteins is considered to be too rapid to be detected in these analyses [49, 50]. As seen previously [51], both H3.3 and H3.1 had fast initial recovery, thought to be due to a loosely bound pool, followed by a slower phase after 30 seconds (Fig 4B and 4C). Interestingly the rate of initial exchange was identical for H3.3 in MCF7 (transformed) and MCF10A (non transformed) cells (Fig 4B), while the rate of initial H3.1 exchange was higher in transformed cells (Fig 4C). It is tempting to speculate that this may indicate that transformed cells may have a larger pool of loosely bound H3.1 than non transformed cells, given that similar results have been seen for H3.1 and H3.3 in pluripotent versus differentiated cells [51]. However, caution must be taken to avoid over interpretation of these data, because we set the lowest fluorescence to zero to normalize for cell to cell variation in the soluble pool. In addition, the MCF7 cells have more cells in S phase, which will have more newly incorporated loose H3.1 protein (S7B Fig), which could also explain these results. Notwithstanding, when we compared the effect of K56Q, K56R on fluorescence recovery we saw no significant difference in the recovery of histone fluorescence in MCF10A or MCF7 cells (Fig 4D and 4E). As such, a mutation mimicking acetylation of H3 K56 is not sufficient to alter chromatin dynamics over the bulk genome.

In summary, we conclude that although a mimic of H3 K56Ac can promote chromatin disassembly from human promoters in the context of transcriptional induction, H3 K56Ac is not used by human cells to drive chromatin disassembly from promoters nor is it required for gene induction at the genes that we studied. Moreover, even the mutation that mimicked acetylation of H3 K56 is not sufficient to drive chromatin disassembly from the bulk genome in the absence of the promoter specific events that occur during gene induction. Moreover, we urge caution when contemplating performing, or interpreting, any analysis in metazoan cells that uses commercial antibodies against H3 K56Ac. The likely reason for the lack of specificity of the H3 K56Ac antibodies is the relative scarcity of this histone modification in metazoans, where less than 0.03% of mammalian histones have H3 K56Ac, which would make more abundant acetylations on the N-terminus of histone H3 more favorable targets for these antibodies. Clearly, when H3 K56Ac is equally abundant to other histone H3 acetylations, as in dot blots, the H3 K56Ac antisera are fairly specific (Fig 1A). As such, we are confident that there will not be specificity issues with the H3 K56Ac antibodies in yeast, because H3 K56Ac is one of the most abundant histone acetylations in yeast. For example, during S phase, all newly-synthesized histones have H3 K56Ac.

We propose that the gold standard for establishing the specificity of antibodies to histone post-translational modifications should be their failure to recognize a histone protein with the relevant amino acid mutated to prevent the modification, in the cellular or organismal context. This *in vivo* scenario allows a true test of antibody specificity, because if the non-specific targets (i.e. the same type of modified amino acid at different locations within the same protein or other proteins) are more abundant in the cell than the specific histone post-translational modification that the antibody is supposed to be recognizing, this may lead to antibody non-specificity. The use of dot blots, where each modified peptide is present at the same abundance, leads to a misleading sense of specificity for low abundance post-translational modifications in physiologically relevant situations.

## Material and Methods

### Cell culture for analysis of Estrogen-responsive genes

MCF7, MCF10 and HEK293 cells were obtained from ATCC. For the gene induction experiments, MCF7 cells plated at 20–30% confluency were washed with PBS three times to ensure complete deprivation of normal FBS that contains hormones including estrogen. After PBS wash, cells were maintained in phenol red-free DMEM with 10% charcoal dextran-stripped FBS (GEMINI BIO-PRODUCTS) for 96hrs to eliminate basal level of estrogen-mediated gene expression. Subsequently the cells were treated with 10μM βeta-estradiol (SIGMA Aldrich), and either total RNA or material for chromatin immunoprecipitation was harvested. Knockdowns for ASF1A and/or ASF1B were achieved using lentiviral vector-mediated shRNA, as described below. Using Fugene, we transfected 72 hour-hormone-depleted MCF7 cells with constructs expressing YFP-tagged histone H3.3 or H3.1 carrying point mutations of lysine 56 residue converted to glutamine (K56Q) or arginine (K56R) that mimic acetylated state or an unacetylated state of lysine residue, and 24hrs later the transfected cells were treated with estrogen to induce gene activation. For the FRET analyses, plasmids carrying YFP-tagged histone H3.3 or H3.1 carrying point mutations of lysine 56 residue converted to glutamine (K56Q) or arginine (K56R) were transfected into MCF7 or MCF10 cells, followed by FRET analysis. The stable cell lines HEK293 H3.1c WT and K56R were generated by integration of a FLAG tagged-plasmid carrying H3.1 that was either wild type or mutated to K56R. The H1299 WT and K56R cell lines were generously provided by Zhenkun Lou and were described previously [47].

### Lentivirus-mediated shRNA

293T cells were transfected with shRNA vectors for silencing of ASF1A/B together with the packaging vectors pCMV-dR8.2 and pCMV-VSV-G. Transfection was performed using Lipofectamine2000 (Life Technologies). The media was once replaced within 18hrs of transfection and the conditioned media was collected 48hrs after transfection. The media was concentrated with Ultra-15 centrifugal filter unit with ultracel-100 membrane (Amicon) at 1500g for 30min at 4°C. The concentrated media containing lentivirus was aliquoted and kept at -80°C until use. For infection, the virus stock was added to MCF7 cells and selected in the presence of 2μg/ml of puromycin for at least 4days to achieve sufficient silencing. Cells were harvested for western blotting. ASF1A antibody (Cell signaling) and ASF1B antibody (Thermo Scientific) was used to check the efficiency of knockdown. A set of bacterial pGIPZ shRNA clones for ASF1A and ASF1B (Open Biosystems) were obtained through core facility of UT MD Anderson Cancer center and the clone capable of silencing ASF1A/B at the highest efficiency was determined.

### ChIP

A 150mm dish plated with MCF7 cells at a confluency of 70–80% was used for each time point. The culture media was aspirated and the plates were washed with PBS twice. After fixation of cells with 25ml of 1% formaldehyde for 15mins at room temperature while gently shaking, glycine was added at a final concentration of 0.125M for quenching. After 5min incubation of glycine quenching, the cells were washed twice with ice-cold PBS. Then cells were harvested with 2ml of lysis buffer (50% glycerol, 50mM Tris-HCl pH7.9, 100mM KCl, 5mM MgCl_2_, 0.05%saponin 1mM DTT, 1mM PMSF, 5mM Na butyrate) containing complete protease inhibitor cocktail (Roche Applied Science). The cell lysate was dounce-homogenized and centrifuged at 4°C, 1300g for 10min. The nuclear pellet was suspended in 1ml of RIPA buffer with protease inhibitor cocktail, 1mM PMSF, and 5mM Na butyrate. The nuclear suspension was sonicated until the insoluble fraction almost disappeared by centrifugation. The shearing of DNA into ~500bp long was later confirmed by extracting genomic DNA. Subsequently the cell suspension was centrifuged at 4°C, 15000g, for 15min and the supernatant fraction was transferred to a fresh tube. The cell lysate was used for 2–3 immunoprecipitations. For immunoprecipitation, the cell lysate was pre-cleared with Dynabeads ProteinA (Life Technologies) for 1hr while the dynabeads that was used to capture antibody used for immunoprecipitation was blocked with BSA and single stranded DNA from salmon testes (SIGMA). The cell lysate was mixed with antibody for immunoprecipitation and dynabeads, and was incubated overnight at 4°C. The dynabeads were washed with RIPA buffer and Wash buffer (100mM Tris-HCl pH8.5, 500mM LiCl, 1% NP-40, 1% Na deoxycholate) and eluted in 80μl of elution buffer (50mM Tris-HCl pH8.0, 0.5mM EDTA, 1% SDS). The eluted DNA was mixed with 400μg of proteinase K (Roche Applied Science) and incubated for 2hrs at 42°C and then for 8hrs at 65°C for reverse-crosslinking followed by 40μg of RNaseA treatment for 2hrs at 37°C. The ChIP DNA was collected with a MinElute PCR purification Kit (QIAGEN). The ChIP DNA was quantitated by real-time PCR with a LightCycler480. A telomeric region was used for normalization. All data shown were normalized to both the relevant input sample at each time point and the telomeric region. An H3 C-term antibody (Abcam) was used to assess H3 occupancy. H3 K56Ac levels are shown normalized to the H3 levels, calculated as above. All experiments were performed three independent times and the average and standard deviation of the results are shown. The student’s unpaired T-test was used to measure statistically significant differences from the data at time zero to later time points for each experimental condition at each genomic location. The primer pairs used for ChIP amplification are given below.

### mRNA quantitation

Steady state transcript levels were measured by RT-PCR. Total RNA was harvested with TRI reagent (Molecular Research Center, Inc.) according to the manufacturer’s protocol. 1μg of total RNA was used for cDNA synthesis. cDNA was synthesized using oligo dT primer provided with Transcriptor First Strand cDNA Synthesis Kit (Roche Applied Science) according to the manufacturer’s protocol. The synthesized cDNA was quantitated by real-time PCR using LightCycler480 (Roche Applied Science). For mRNA quantitation, the amount of cDNA was normalized to that of the housekeeping gene GAPDH.

### Real time PCR primers

For ChIP:

pS2-distal-ERE;

Forward 5’- CTGGGTGACAGGAAAGAAGC-3’

Reverse 5’- CATTCTGGAAGGGACACACA -3’

pS2-proximal-ERE

Forward 5’- GCTTAGGCCTAGACGGAATGGGC-3’

Reverse 5’- CCAGGTCCTACTCATATCTGAGAG-3’

GREB1-distal-ERE

Forward 5’- GAGCTGACCTTGTGGTAGGC-3’

Reverse 5’- GGTTTTTAAGCAGCCAGCAG-3’

GREB1- proximal-ERE

Forward 5’- TTGTTGTAGCTCTGGGAGCA-3’

Reverse 5’- CAACCAGCCAAGAGGCTAAG-3’

PR-205

Forward 5’- AAAGAGAGTGAGTCATTTGTG-3’

Reverse 5’- CAGGAGATCCGTGAGTTC-3’

PR-221

Forward 5’- GGGAAATTGCCTCTCCTCACTTTG-3’

Reverse 5’- CCAAGGATTAGGGCAGTTCAGAAG-3’

PR-95

Forward 5’- CAG GCT ATT TCT CAG GTC AG-3’

Reverse 5’- GAC AAA CAC ATT CCC AAA CC-3’

PR+4

Forward 5’- TTGGTTCTGCTTCGGAATCTG-3’

Reverse 5’- CCTCCTCTCCTCACTCTTGG -3’

For mRNA quantitation:

GAPDH

Forward 5’- CGAATTTGGCTACAGCAACAGGGT

Reverse 5’- TGAGGGTCTCTCTCTTCCTCTTGT

GREB1 *

Forward 5’- GGCAGGACCAGCTTCTGA

Reverse 5’- CTGTTCCCACCACCTTGG

PGR

Forward 5’- GCTCCTCATTTCTGAGTGGGAAAG

Reverse 5’- CCCAGGCATACACAGATGAAAGGA

TFF1 *

Forward 5’- TTGTGGTTTTCCTGGTGTCA

Reverse 5’- CCGAGCTCTGGGACTAATCA

* Primers for GREB1 and TFF1 were previously used [52]

Telomeric region on chromsome 2

Forward 5’- ACATGGGAGAGTGAAGGTGGGTTA-3’

Reverse 5’- TGTAGGGACTTGTGCTACCATCTC-3’

ASF1A

Forward 5’-CTTGCAGCTAAGCAAGACAGCCAT-3’

Reverse 5’-CTCAGAATCCATGTTTAGACAAATGCCC-3’

ASF1B

Forward 5’-TAGGACCAGGGTGATTTCAAGCCA-3’

Reverse 5’-ATCACAACAGCATCCACATGGCAG-3’

### Dot blots

Lyophilized peptides were rehydrated in 1 x PBS at a 300 μM concentration and then diluted 10 fold, prior to spotting 10 μl onto activated PVDF membrane. The membrane was air-dried and then stained with amido black to verify the presence of the peptides. The membranes were washed in TBST and then blocked in 3% non-fat milk in TBST. The blots were incubated in primary antibodies overnight at 4°C. The blots were washed and probed with HRP conjugated secondary antibodies at room temperature for 1 hr.

### Western blots

Acid extracted histones were separated by SDS-PAGE, probed with the indicated antibodies in each figure. Where necessary, the secondary antibody IRDye^®^ 680RD Goat anti-Rabbit IgG (H + L) multiplexed with the IRDye^®^ 800CW Goat anti-Mouse IgG (H + L) was used for the 2-color detection method by the Odyssey LI-COR imaging system. The H3 K56Ac antibodies used in this and other experiments are: Epitomics monoclonal Ab (2134–1), Epigentek polyclonal Ab (A-4026-050), Cell Signaling polyclonal Ab (4243S), Upstate polyclonal Ab (07–677), Active Motif polyclonal Ab (39281), Active Motif monoclonal Ab (61061). The other antibodies used are: N-terminal histone H3 mAb (Active Motif 39763), C-terminal histone H3 pAb (Abcam ab1791), IRDye 680 Goat anti-rabbit IgG (LICOR 926–32221) and IRDye 800CW Goat anti-mouse IgG (LICOR 926–32210).

### Peptide competition assay

Biotinylated peptides (4 μg total) were incubated with 400 microlitres of antibody for 45 min at room temperature (RT), followed by capturing the peptides on strepdavidin agarose. Samples were centrifuged and 300 μl of each supernatant was used for western analysis of acid-extracted histones from HeLa cells, as described above.

### Immunohistochemistry (IHC)

Slides containing paraffin embedded breast cancer tissue samples fixed in 10% neutral buffered formalin were obtained from the MD Anderson Pathology core and IHC method was optimized in assistance with the Pathology core. In short, the sections of the tissues were deparaffinized with xylene and rehydrated in a graded series of ethanol (two 100% ethanol wash, followed by 95% ethanol, 70% ethanol, 50% ethanol, 30% ethanol, followed by water). Antigen retrieval was performed using heat-induced method following standard procedure. After blocking non-specific binding, the sections were incubated with primary antibody at indicated dilution (overnight, 4°C, humidified chamber) followed by incubation with HRP-conjugated secondary antibody (1 hr, room temperature). For signal detection, DAB substrate kit (Vector Laboratories) was used followed by counterstaining with Mayer’s Hematoxylin (Sigma).

### Fly work

The genotype of the fly line used in this study is *yw*,*Ubx-FLP; Df(2L)His*^*C*^ *FRT40A/ GFP FRT40A; 6xHisGU* ^*VK33*,*27*^ *H3K56R/ 6xHisGU* ^*VK33*,*27*^ *H3K56R*. Dissections and staining of wing discs were performed according to standard protocols. Primary antibodies used in this study were used at 1:100–1:500 dilution. The secondary antibody used was Cy3 (1:600, Jackson Immunoresearch). More than 15 discs were analyzed for each mutant. Images were obtained using a FV1000 Olympus confocal and all images represent Z-stacks of the disc proper portion of the imaginal disc.

### FRAP

Cells were imaged using a 3i Marianas Spinning Disk Confocal microscope equipped with an Evolve 10 MHz Digital Monochrome Camera (Photometrics, Tuscon, AZ USA) for the photobleaching experiments and fluorescent image acquisitions. Photobleaching and quantitation was performed as described previously [51]. Images were collected every 500ms. Image analysis was performed with MetaMorph imaging software.

## Supporting Information

S1 Fig ChIP analysis of H3 and H3 K56Ac showing that levels do not change at a control region. The data shown is that for the telomeric control region used as an internal normalization control for the experiments shown in Fig 1B.
(TIFF)

S2 Fig ChIP analysis of H3 and H3 K56Ac upon knockdown of ASF1 proteins in human cells. ChIP analysis of histone H3 occupancy at the indicated time points after estradiol addition from the same time course shown in Fig 1C and 1D, following knockdown using scrambled shRNA, shRNA of ASF1A, ASFB and ASF1A+B, at the indicated EREs in the *TFF1*, *GREB1*, and *PGR* promoters. Each data point was normalized to the input and a telomeric control region at the same time point. Below each H3 ChIP is shown a ChIP analysis of histone H3 K56Ac levels normalized to H3 occupancy from the same experiments. Each data point was normalized to the input and a telomeric control region at the same time point. Shown are the average and standard deviation of three independent experiments. * indicates significant changes from time 0, p<0.05 measured by the Student’s unpaired t-test.
(TIFF)

S3 Fig Analysis of TFF1 transcription upon ASF1 knockdown. Real time PCR analysis of cDNA performed as in Fig 1B, with the indicated knock downs. The analysis performed here was from the same experiment as Fig 1C and 1D.
(TIFF)

S4 Fig Additional tests of specificity of the H3 K56Ac antibodies. A. Peptide competition as described in Fig 2A. This analysis was done in parallel with the one in Fig 2A, so the same loading control is shown. B. Western analysis of FLAG-tagged histone H3 with the indicated mutation, using the indicated antibodies.
(TIFF)

S5 Fig Additional tests of specificity of the H3 K56Ac antibodies, part II. A. MCF7 cells were transfected with empty vector or vector encoding H3.3-YFP that was wild type or had K56 mutated to R or Q, as indicated. 75 micrograms of total protein extract was loaded for each lane, and western blotted with the indicated antibodies, followed by detection with infrared antibodies on a Licor Odyssey machine. B. IHC analysis of breast cancer tissue using either no primary antibody or the indicated dilutions of the indicated antibody. IHC staining was as described for Fig 2C.
(TIFF)

S6 Fig Overview of the fly system used to delete the endogenous histone gene locus, HisC. Only chromosome II is shown. Orange triangles indicate FRT recombination sites. Induction of a tissue specific flippase causes recombination to swap the left arm of 2L between chromosomes. The following mitoses result in three types of cells (i) those that are very green (with two copies of GFP) and have two copies of wild type HisC, labeled wild type, (ii) cells that have no HisC locus and no GFP labeled mutant (iii) cells that have one HisC locus and one copy of GFP, labeled Het.
(TIFF)

S7 Fig **A. Overview of the FRAP procedure B. Flow cytometry analysis of DNA content of cells from the same experiments shown in** Fig 4.
(TIFF)
