# Supplementary figures and images for: The Commercial Antibodies Widely Used to Measure H3 K56 Acetylation Are Non-Specific in Human and Drosophila Cells
Source: PLoS One. 2016 May 17;11(5):e0155409. doi: 10.1371/journal.pone.0155409 (PMC4871326; doi:10.1371/journal.pone.0155409)

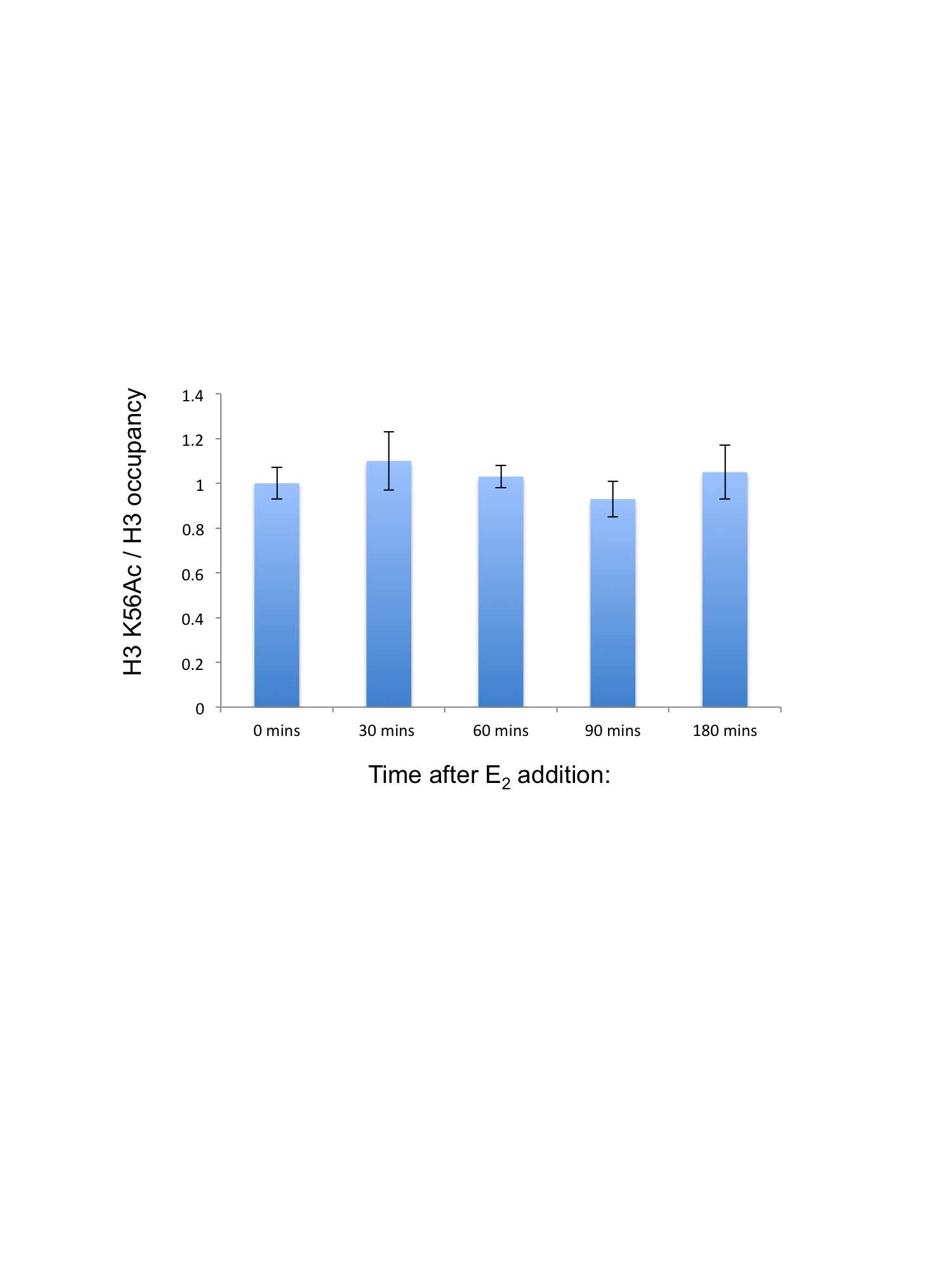

Supplement: S1 Fig — The data shown is that for the telomeric control region used as an internal normalization control for the experiments shown in Fig 1B. (TIFF) [file pone.0155409.s001.tiff]

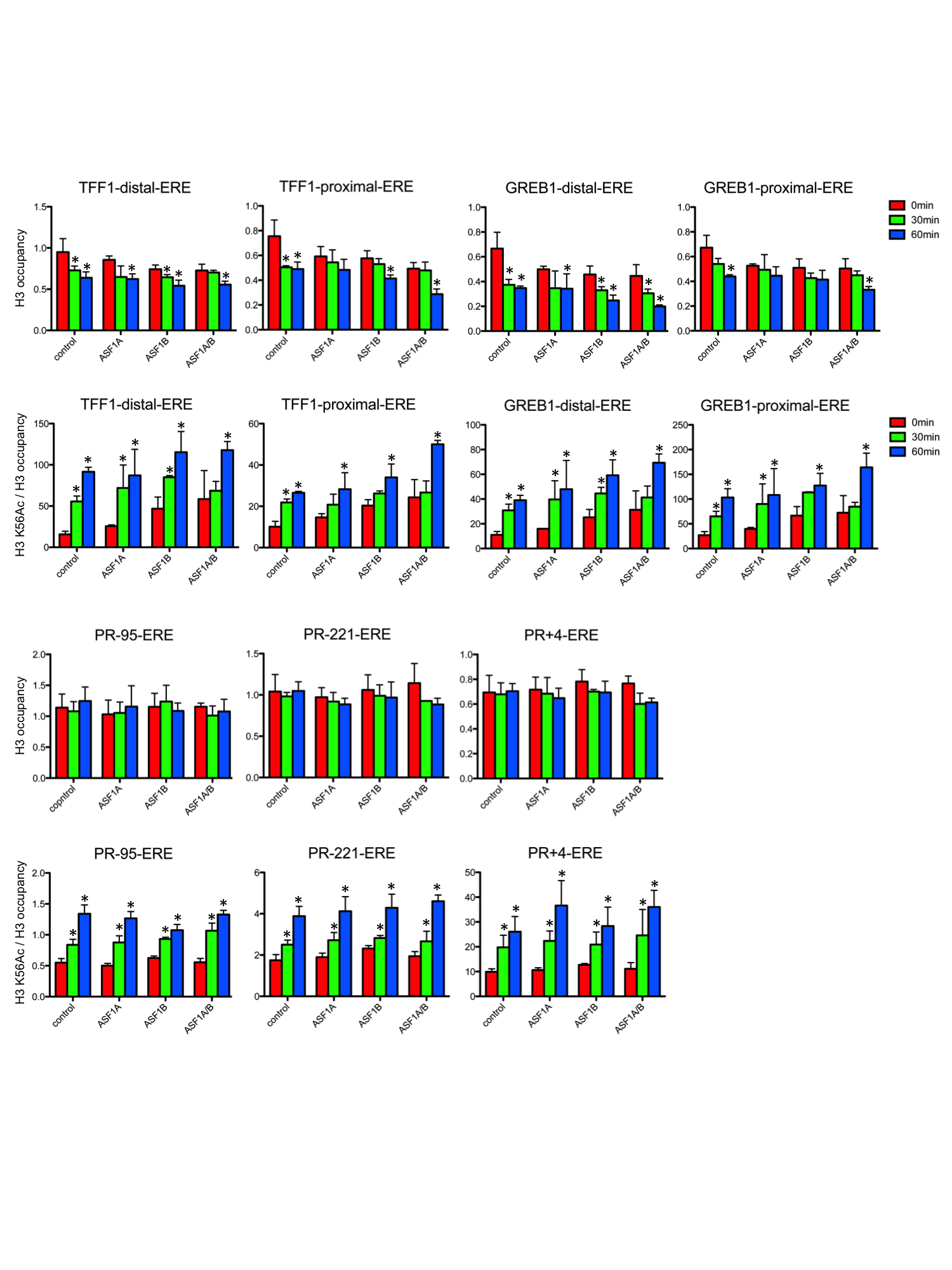

Supplement: S2 Fig — ChIP analysis of histone H3 occupancy at the indicated time points after estradiol addition from the same time course shown in Fig 1C and 1D, following knockdown using scrambled shRNA, shRNA of ASF1A, ASFB and ASF1A+B, at the indicated EREs in the TFF1, GREB1, and PGR promoters. Each data point was normalized to the input and a telomeric control region at the same time point. Below each H3 ChIP is shown a ChIP analysis of histone H3 K56Ac levels normalized to H3 occupancy from the same experiments. Each data point was normalized to the input and a telomeric control region at the same time point. Shown are the average and standard deviation of three independent experiments. * indicates significant changes from time 0, p<0.05 measured by the Student’s unpaired t-test. (TIFF) [file pone.0155409.s002.tiff]

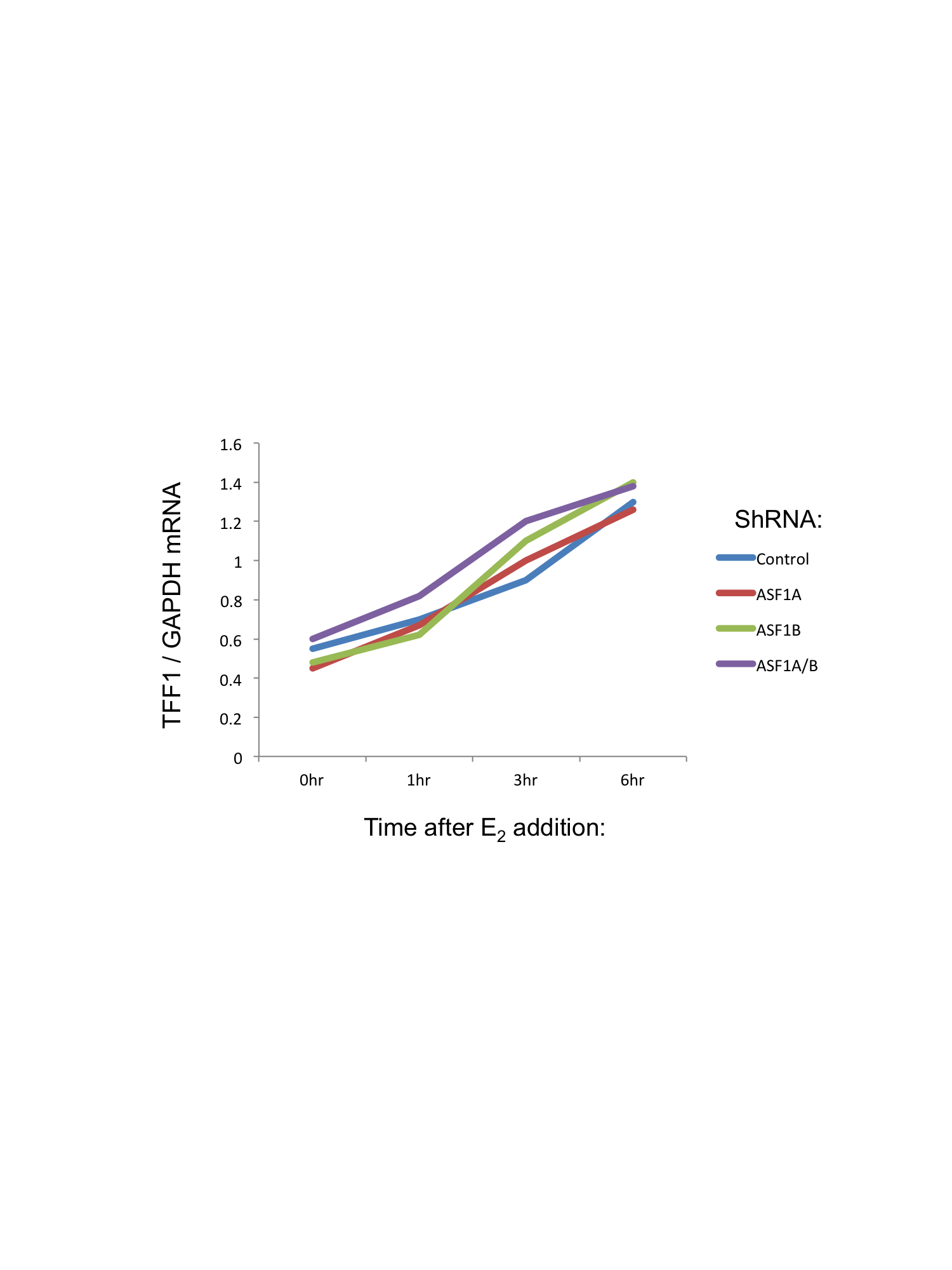

Supplement: S3 Fig — Real time PCR analysis of cDNA performed as in Fig 1B, with the indicated knock downs. The analysis performed here was from the same experiment as Fig 1C and 1D. (TIFF) [file pone.0155409.s003.tiff]

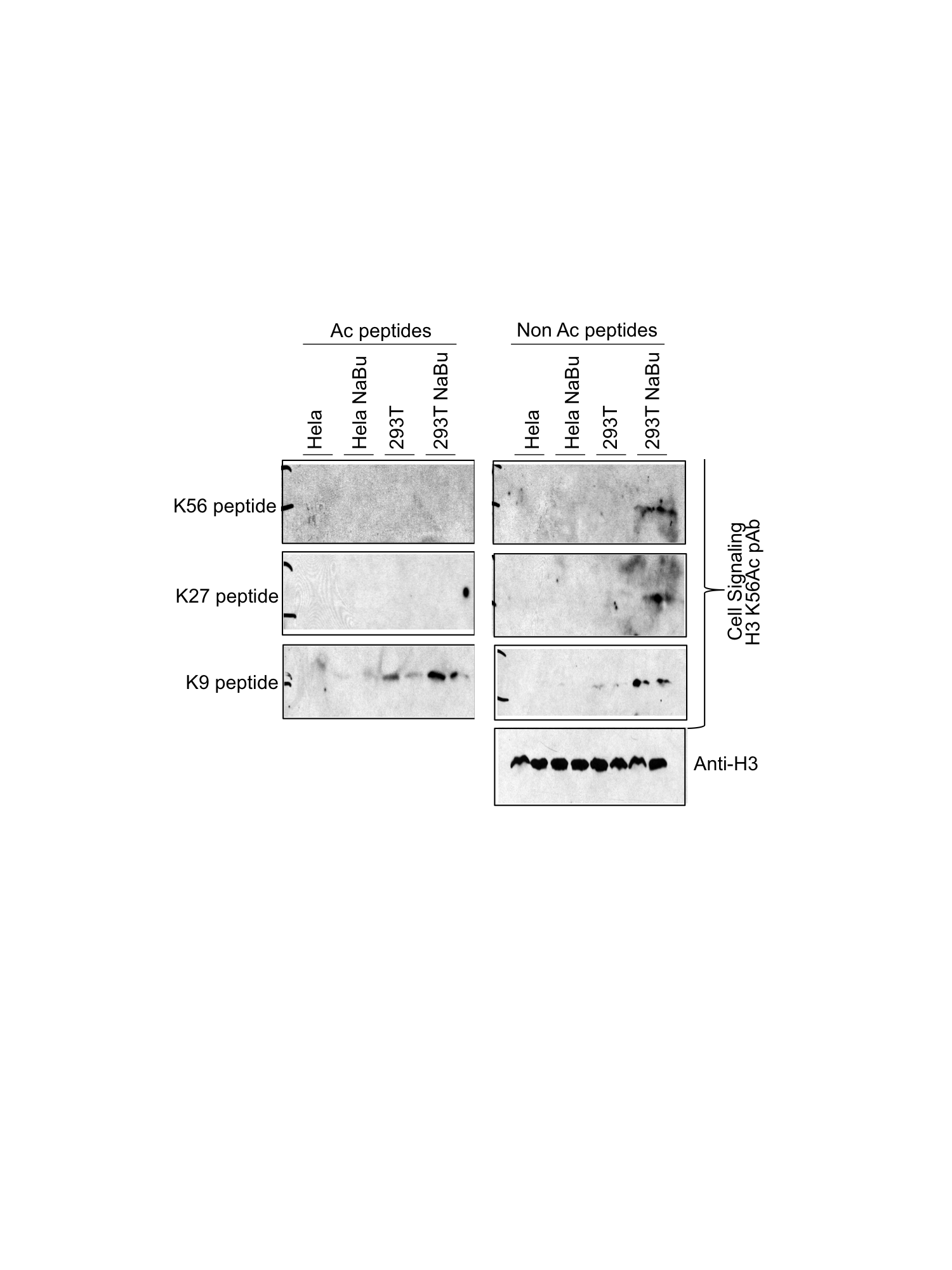

Supplement: S4 Fig — A. Peptide competition as described in Fig 2A. This analysis was done in parallel with the one in Fig 2A, so the same loading control is shown. B. Western analysis of FLAG-tagged histone H3 with the indicated mutation, using the indicated antibodies. (TIFF) [file pone.0155409.s004.tiff]

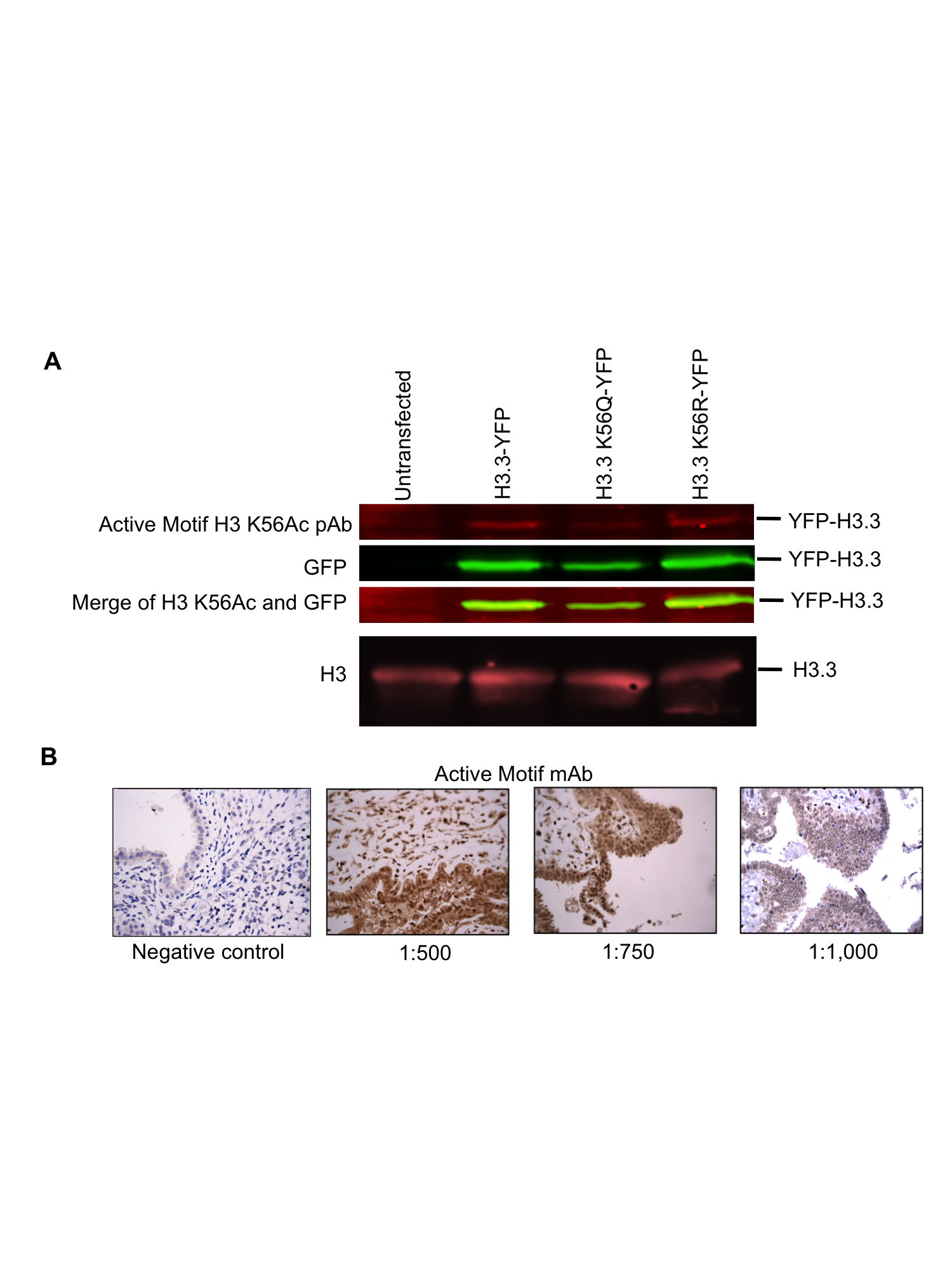

Supplement: S5 Fig — A. MCF7 cells were transfected with empty vector or vector encoding H3.3-YFP that was wild type or had K56 mutated to R or Q, as indicated. 75 micrograms of total protein extract was loaded for each lane, and western blotted with the indicated antibodies, followed by detection with infrared antibodies on a Licor Odyssey machine. B. IHC analysis of breast cancer tissue using either no primary antibody or the indicated dilutions of the indicated antibody. IHC staining was as described for Fig 2C. (TIFF) [file pone.0155409.s005.tiff]

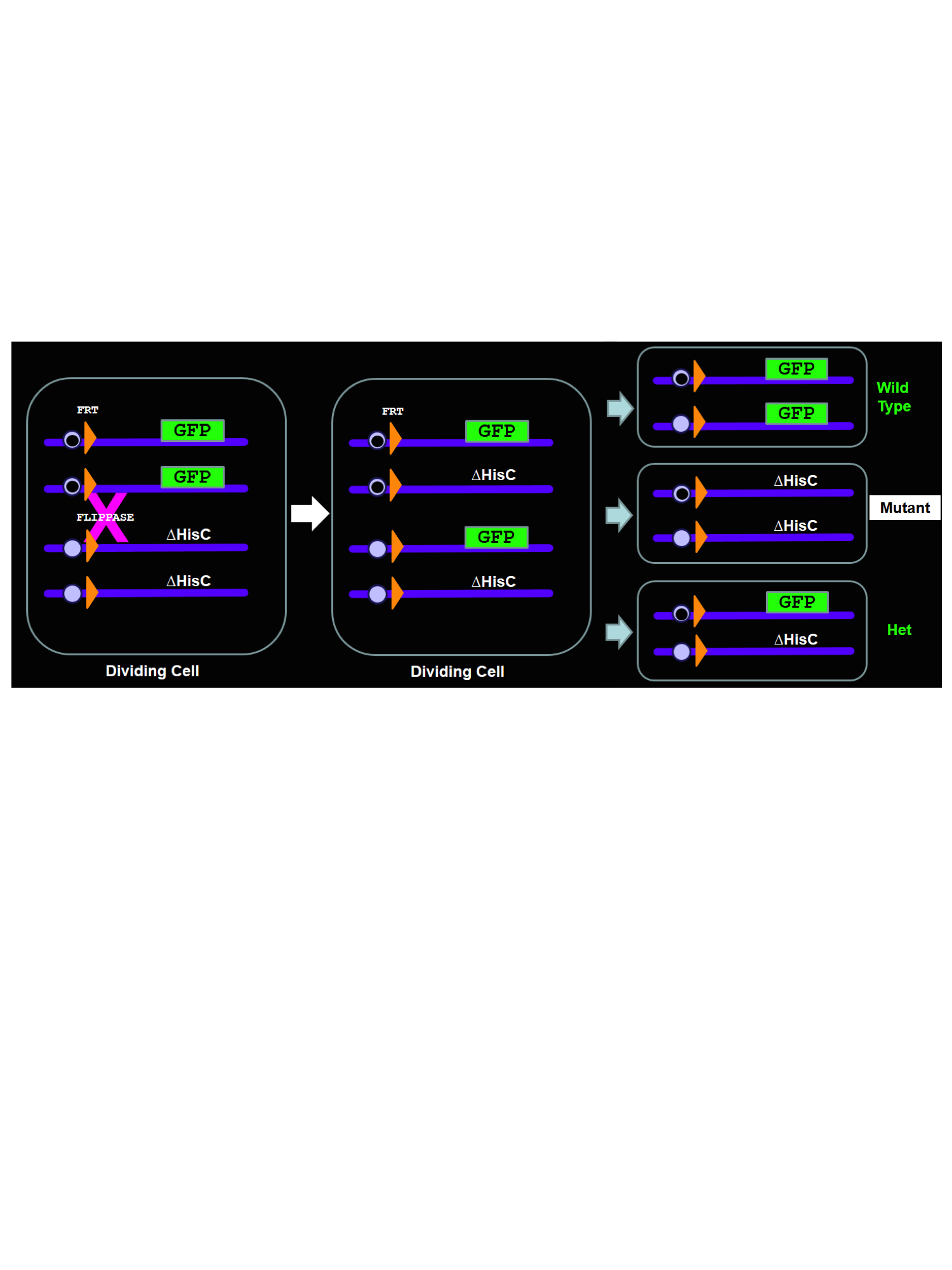

Supplement: S6 Fig — Only chromosome II is shown. Orange triangles indicate FRT recombination sites. Induction of a tissue specific flippase causes recombination to swap the left arm of 2L between chromosomes. The following mitoses result in three types of cells (i) those that are very green (with two copies of GFP) and have two copies of wild type HisC, labeled wild type, (ii) cells that have no HisC locus and no GFP labeled mutant (iii) cells that have one HisC locus and one copy of GFP, labeled Het. (TIFF) [file pone.0155409.s006.tiff]

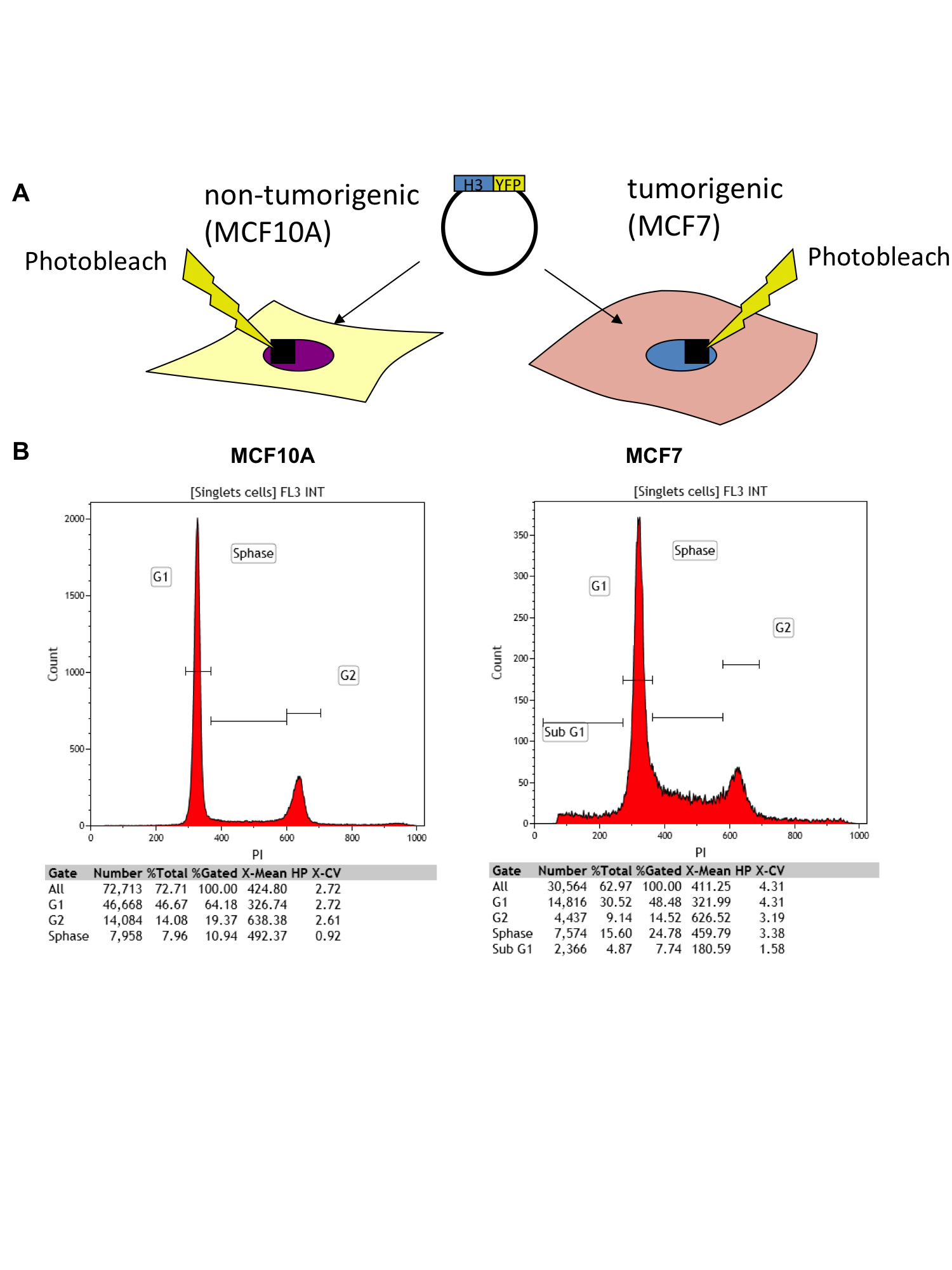

Supplement: S7 Fig — A. Overview of the FRAP procedure B. Flow cytometry analysis of DNA content of cells from the same experiments shown in Fig 4. (TIFF) [file pone.0155409.s007.tiff]
